# Supplementary material for: Effects of Cannabidiol on Innate Immunity: Experimental Evidence and Clinical Relevance
Source: Int J Mol Sci. 2023 Feb 4;24(4):3125. doi: 10.3390/ijms24043125 (PMC9964491; doi:10.3390/ijms24043125)
Supplement: Supplementary file 1 [file ijms-24-03125-s001.zip › ijms-2161624-supplementary.pdf]

Table S1. Studies including in the review concerning CBD effects on innate immunity.

Source	Models	Function investigated	Main results	CBD effect	Reference
**Macrophages / monocytes**					
RAW-264.7 monocytes/macrophages cell line.	Cells were treated with UDP, ATP IFN-γ, LPS, dexamethasone to induce phagocytosis, then with CBD (10 μM) and incubated with beads.	• Evaluation of phagocytosis.	• Increase of phagocytosis.	↑	[107]
Swiss male mice (18-20 g) macrophages.	Peritoneal exudate cells treated with CBD (from 0.001 to 1 μM) and LPS.	• Evaluation of cytokines (IL-10 and 12) release.<br>• Evaluation of chemotaxis fMLP-induced.	• Decrease of IL-10 release and increase of IL-12 release.<br>• Decrease of chemotaxis fMLP-induced.	↑↓	[122]
Swiss male mice (18-20 g).	Mice inoculated i.p. with thioglycolate and injected with CBD (15-30 mg/kg). Isolated macrophages were treated with LPS.	• Evaluation of cytokines (IL-10 and 12) release by macrophages.	• Decrease of IL-10 release and increase of IL-12 release.	↑	[122]
Monocytes from healthy subjects.	Human peripheral monocytes freshly isolated or precultured, treated with CBD and doxorubicin to induce apoptosis.	• Evaluation of apoptosis.	• Increase of apoptosis of freshly isolated monocytes.	↓	[127]
THP-1 monocytes cell line.	Cells treated with CBD.	• Evaluation of ROS production.<br>• Evaluation of apoptosis.	• Increase of ROS generation.<br>• Increase of apoptosis.	↓↑	[124]
Monocytes from healthy subjects	Monocytes treated with CBD (16 uM).	• Evaluation of apoptosis.<br>• Evaluation of ROS generation.	• Increase of apoptosis<br>• Increase of ROS generation.	↓↑	[125]
RAW 264.7 macrophages cell line.	Cells were incubated CBD (5 μM) and LPS+ATP.	• Evaluation of NF-κB activation.<br>• Evaluation of NLRP3 inflammasome activation.<br>• Evaluation of cytokines (IL-1β, TNF-α, and MCP-1) expression.	• Inhibition of NF-κB activation.<br>• Suppression of activation of NLRP3 inflammasome.<br>• Reduction of IL-1β, TNF-α, and MCP-1 mRNA levels.	↓	[116]

6-week-old male C57BL/6J mice.	Mice with liver inflammation HFC-induced were hand-fed with CBD (5 mg/kg per day).	• Evaluation of hepatic macrophage infiltration.	• Decrease of macrophages infiltration in liver.	↓	[116]
U937 and RAW264.7 macrophages cell line.	Cells were treated with LPS and CBD.	• Evaluation of cytokines (IL-8, IL-16, IL-32, IFN-γ, IL-1A, IL-6, IL-27, I-TAC, M-CSF, MCP-1, CCL2, CCL5, TNF-α, MIF) levels in U937.<br>• Evaluation of G-CSF, GM-CSF in RAW264.7.<br>• Evaluation of ROS production in RAW264.7.	• Reduction in LPS-induced NF-κB activity, and IL-8, and MCP-1 release.<br>• Increased serpin E1, CXCL1, IL-6, MIF, IFN-γ, MCP-1, RANTES, and TNF-α production<br>• Reduction in MCP-1/CCL2, CCL5, eotaxin, and IL-2 production.<br>• Increase of ROS generation.	↑↓	[123]
THP-1 monocytes cell line.	Cell stimulated with LPS+nigericin and treated with CBD (0.1, 1, and 10 μM).	• Evaluation of cytokines (IL-1β, TNF-α) release.	• Decrease of IL-1β release.	↓	[119]
6-week-old female C57BL/6 mice.	Murine model of EAE treated with CBD (20 mg/kg).	• Evaluation of cytokines (CXCL9, CXCL10 and IL-1β) mRNA expression levels in macrophages.<br>• Evaluation of macrophages infiltration into CNS.	• Reduction of IL-1β and CXCL10 mRNA expression levels.<br>• Reduction of macrophages infiltration.	↓	[103]
Monocytes from healthy subjects.	Human primary monocytes activated through TLR 1–9 and treated with CBD (0.5–10 μM).	• Evaluation of cytokines (IL-4, IL-2, IP-10, IL-1β, TNFα, MCP-1, IL-17a, IL-6, IL-10, IFNγ, IL-12p70, IL-8, and TGF-β1) secretion.	• Inhibition of IL-1β and IL-6.	↓	[121]
THP-1 monocytes cell line.	Cells treated with LPS and CBD (from 5 to 100 uM).	• Evaluation of cytokines (IL-1β, IL-6, and IL-10, MCP-1, VEGF, RANTES) expression.<br>• Evaluation of mTOR, endothelial NOs, COX2 expression.	• Reduction of TNF-a, RANTES, IL-1β and -6 expression.<br>• Reduction in levels of phosphorylated m-TOR Ser 2448.<br>• Reduction of endothelial NOs.<br>• Induction of COX2.	↑↓	[120]

RAW 264.7 macrophages cell line.	Cells were stimulated with LPS and treated with CBD.	• Evaluation of cytokines (TNF-α and Il-1β) release.	• inhibition of TNF-α and IL-1β.	↓	[117]
5/8-week-old male Wistar rats (150-250 g).	Rats with pulmonary hypertension-MCT induced, injected *i.p.* with CBD (10 mg/kg)	• Number of infiltrated macrophages in lung.	• Decrease of number of macrophages infiltrated in lung.	↓	[126]
Monocytes from healthy subjects.	Monocytes differentiated in macrophages, treated with CBD (10 μM).	• Evaluation of transcriptional cell profile.<br>• Evaluation of autophagy process.	• Inhibition of cGAS-STING-mediated activation of type I Interferon response genes<br>• Upregulation of autophagy receptor p62/SQSTM1	↓↑	[129]
THP monocytic cell line.	Cells treated with CBD (10 μM).	• Evaluation of transcriptional cell profile.<br>• Evaluation of autophagy process.	• Inhibition of cGAS-STING-mediated activation of type I Interferon response genes<br>• Upregulation of autophagy receptor p62/SQSTM1	↓↑	[129]
8- to 11-week-old female and male and 8-month-old C57BL/6 female mice macrophages.	Exudate macrophages were treated with CBD (5 μM) and stimulated with LPS.	• Evaluation of NO production.<br>• Evaluation of cytokines (IL6, TNF-α, CXCL2, G-CSF) expression.<br>• Evaluation of MHCII expression.	• Inhibition of NO secretion.<br>• Inhibition of IL-6, TNF-α, CXCL2 expression.<br>• Decrease of MHCII expression.	↓	[118]
8- to 11-week-old female and male and 8-month-old C57BL/6 female mice.	Murine colitis DSS-induced, injection i.p of CBD (5 mg/kg).	• Evaluation of Macrophages infiltration in colon.	• Decrease of cell infiltration in colon.	↓	[118]
**Mast cells**					
RBL-2H3 cells line.	RBL-2H3 mast cell activated with IgE receptor, incubated with HSA antigen and treated with CBD (1-10 μM).	• Beta-hexosaminidase cells release.<br>• Evaluation of Ca^{2+} intracellular levels.	• Enhancement of beta-hexosaminidase cells release.<br>• Rise of intracellular Ca^{2+}	↑	[89]
Male Swiss OF1 mice (30–40 g) mast cells.	Mice model of intestinal inflammation, injection i.p. of CBD (10 mg/kg) before LPS treatment.	• Release of mast cells chymase and MMP9.	• Inhibition of mast cells chymase and MMP9 release.	↓	[90]
**Astrocytes**					

Male ddY mice (25-35 g).	Mouse were subjected to middle cerebral artery occlusion and treated with of CBD (3 mg/kg).	• Evaluation of MPO activity in brain.	• Reduction of MPO activity.	↓	[94]
5/7-week-old male Swiss albino mice (30-40 g).	Mice were subjected to bilateral common carotid artery occlusion and treated with CBD (3-30 mg/kg)	• Evaluation of GFAP immunoreactivity in brain.	• Reduction in GFAP expression.	↓	[95]
HBMEC and human astrocytes.	BBB permeability model by co-cultures of HBMEC and human astrocyte in oxygen-glucose deprivation conditions, treated with CBD (10 μM).	• Evaluation of BBB permeability.<br>• Evaluation of LDH release.<br>• Evaluation of VCAM-1 and VEGF levels.	• Reduction BBB permeability.<br>• Decrease VCAM-1 levels.<br>• Increased VEGF levels.	↓	[92]
Rat CTX-TNA2 astrocytes.	Astrocytes were treated with H_2O_2 and CBD (1μM).	• Evaluation of ROS production.<br>• Evaluation of proliferation.<br>• Evaluation of apoptosis.	• Reduction in ROS production.<br>• Increase in proliferation.<br>• Reduced early apoptosis.	↓	[93]
2/4-day-old C57BL/6J mice astrocytes.	Astrocytes incubated with CBD (5-10 μM) followed by LPS.	• Evaluation of IL-6 and TNF-α release.<br>• Evaluation of Nf-κB and STAT3 pathways.	• Inhibition of IL-6 release but not TNF-α.<br>• Inhibition of NF-κB and STAT3 phosphorylation.	↓	[105]
6-week-old C57BL/6J mice.	Mice were injected i.p. with CBD (10mg/kg or 75mg/kg) followed by LPS.	• Evaluation of mRNA expression of IL-1β, TNF-α and IL-6 by astrocytes.	• Inhibition of mRNA expression of IL-1β and TNF-α but not IL-6.	↓	[105]
**Oligodendrocytes**					
12-day-old Sprague Dawley rat oligodendrocytes.	Oligodendrocytes incubated with CBD (0,1-10 μM).	• Evaluation of ROS generation.<br>• Evaluation of Ca^{2+} release.<br>• Evaluation of apoptosis pathway.	• Increase of ROS production.<br>• Increase of Ca^{2+} release.<br>• Increase of apoptosis.	↓↑	[110]
0/2-days-old Wistar rats OPC.	OPC were treated with LPS/IFNγ/H_20_2/ Tunicamycin in presence of CBD (1 μM).	• Evaluation of ROS generation.<br>• Evaluation of apoptosis.	• Decrease of ROS production.<br>• Decrease of caspase 3 expression.<br>• Decrease of apoptosis ER stress-induced.	↓↑	[111]
**Microglia cells**					

8/10-week-old male C57BL6 mice microglia.	Microglial cells were treated with UDP, ATP, IFN-γ, LPS, dexamethasone to induce phagocytosis, then with CBD (10 μM) and incubated with beads.	• Evaluation of phagocytosis.	• Increase of phagocytosis.	↑	[107]
BV-2 microglial cells line.	Microglial cells were treated with UDP, ATP IFN-γ, LPS, dexamethasone to induce phagocytosis, then with CBD (10 μM) and incubated with beads.	• Evaluation of phagocytosis.	• Increase of phagocytosis.	↑	[107]
HAPI cells line.	Microglial cells were treated with UDP, ATP IFN-γ, LPS, dexamethasone to induce phagocytosis, then with CBD (10 μM) and incubated with beads.	• Evaluation of phagocytosis.	• Increase of phagocytosis.	↑	[107]
BV-2 mouse microglial cell line.	BV-2 microglial cells were treated with LPS and CBD (10 μM).	• Evaluation of IL-1β, IL-6, and IFN-β production and release.	• Reduction of IL-1β, IL-6, and IFN-β production and release.	↓	[101]
BV-2 mouse and N-13 microglial cells line.	BV-2 and N-13 microglial cells treated with CBD (0,1 μM).	• Evaluation of microglia migration.<br>• Evaluation of NO generation.<br>• Evaluation of IL-6 and TNF-α gene expression.<br>• Evaluation of intracellular Ca^{2+} levels.	• Increase in microglial cells migration.<br>• Inhibition of NO generation.<br>• Increase in IL-6 gene expression.<br>• Reduction in intracellular Ca^{2+} levels.	↑↓	[104]
0-day-old BALB/c mice microglial cells.	Microglial cells treated with CBD (16 μM).	• Evaluation of apoptosis pathway.	• Increase in caspase 8 and 29 activation.	↓	[106]
6-week-old female C57BL/6 mice.	Murine model of EAE treated with CBD (20 mg/kg).	• Microglia Transcriptional Profile.	• Shift in the transcriptional profile to anti-inflammatory profile in microglia within CNS.	↓	[103]
6-week-old C57BL/6J mice microglial cells.	Microglial cells incubated with CBD (10 μM) followed by LPS.	• Evaluation of IL-6 and TNF-α release.	• Inhibition of IL-6 release but not TNF-α.	↓	[105]
6-week-old C57BL/6J mice.	Mice were injected i.p with CBD (10mg/kg or 75mg/kg) followed by LPS.	• Evaluation of mRNA expression of IL-1β, TNF-α and IL-6 by microglia.	• Inhibition of mRNA expression of IL-1β and TNF-α but not IL-6.	↓	[105]

7/9-day-old Wistar rat microglia.	Microglia isolated from Hippocampi slices treated with kainite acid and incubated with CBD (0.1–10 μM).	• Evaluation of microglia activation.	• Inhibition of microglia activation.	↓	[99]
APP/PS1 female mice microglia.	Microglial cells treated with CBD (5 μM).	• Evaluation of Phagocytic Receptors Expression TRPV2, TREM2, GPR34, CR3.	• Increase of mRNA expression of TRPV2 and TREM2.	↑	[108]
**Polymorphonuclear leukocytes**					
Female mice C57BL/6.	PMNs were treated with CBD (6 μM).	• Evaluation of ROS production.	• Suppression of Zymosan-induced ROS.	↓	[163]
PMN from human healthy subjects.	PMNs were activated with fMLP and treated with CBD (0,00001 to 1 μM).	• Evaluation of chemotaxis.	• Decreased chemotaxis.	↓	[153]
Male ddY mice (25–35 g).	Ischemic brain damage was induced by focal I/R injury, CBD (3mg/kg) was administered i.p. immediately before and 3 h after cerebral ischemia.	• Evaluation of PMNs infiltration in brain.	• Decreased PMNs infiltration.	↓	[94]
Male Wistar rats (250–350 g).	Experimental periodontitis was induced by a ligature placement around both mandible first molars before CBD treatment (5 mg/kg) administered i.p. daily for 30 days.	• Evaluation of PMNs infiltration in gingival tissue.<br>• Evaluation of PMN activity in gingival tissue.	• Reduced PMNs infiltration and MPO activity in gingival tissue.	↓	[157]
PMN from human healthy subjects.	PMNs were pre-treated with 1 μM CBD and activated by TNF-α.	• Evaluation of PMNs adhesion.	• Reduced PMNs adhesion.	↓	[155]
Male mice C57BL/6J.	Mouse model of hepatic I/R injury, CBD treatment (3 and/or 10 mg/kg) was injected i.p 60 minutes before the occlusion of hepatic artery.	• Evaluation of PMNs infiltration and activity in liver.	• Reduced PMNs infiltration and MPO activity in liver.	↓	[155]
8-week-old male mice C57BL/6 (20-25 g).	Acute lung injury was induced by intranasal instillation of LPS, CBD (0.3, 1.0, 10, 20, 30, 40, and 80 mg/kg) was administered i.p. 60 minutes before lung injury.	• Evaluation of PMNs Infiltration and activity in lung.	• Decreased PMNs infiltration and MPO activity in lung.	↓	[36]

5/8-week-old female mice C57BL/6.	Pulmonary inflammation was induced by intranasal instillation of LPS, CBD (75 mg/kg) were co-administered by oral gavage.	• PMN count in lung.<br>• Evaluation of TNF-α; IL-6, IL17a; IL-23 and G-CSF mRNA expression levels.	• Enhanced PMNs infiltration in lung.<br>• Increase in mRNA expression levels for TNF-α; IL-6, IL-23 and G-CSF.	↑	[161]
Male adult Dunkin-Hartley guinea pigs (250-350 g).	Airway inflammation was induced by exposition to aerosolized solution of LPS or intranasal instillation of TNF-α, CBD (10 mg/ml) was nebulized 30 minutes before the injury	• PMNs infiltration in airways.	• No effects of CBD on PMNs infiltration in airways.	↔	[162]
PMN from human healthy subjects.	PMNs were activated with PMA before 1μM CBD treatment.	• Evaluation of ROS production.	• Reduction in PMA-induced ROS production.	↓	[159]
10/12-week-old female mice C57BL/6J (25 g).	Alcohol induced liver steatosis; CBD (10 mg/kg) was administered i.p. for 11 days during alcohol exposure.	• Evaluation of PMNs infiltration and activity in liver.<br>• Evaluation of MPO.<br>• Evaluation of ROS production.	• Decreased in PMNs infiltration<br>• Decreased MPO activity in liver.<br>• Decrease in PMNs oxidative burst.	↓	[159]
9/11-week-old male ICR mice.	Acute kidney injury was induced by bilateral renal I/R injury, CBD (10 mg/kg) was administered i.p. 10 minutes before removal of vascular clamps.	Evaluation of PMNs polarization (N1/N2 phenotype ratio).	• Increased N2 PMNs levels in kidney.<br>Decreased N1 PMNs levels in kidney.	↓	[167]
Male BALB/c mice (20-30 g) and CB2R -/- mice.	Model of corneal hyperalgesia, CBD was administered topically post cauterization.	Evaluation of PMNs infiltration in cornea.	Reduced PMNs infiltration in cornea.	↓	[158]
PMN from human healthy subjects.	PMNs were activated with fMLP and IL-8 and co-treated with CBD (0.01-10 μM).	• Evaluation of chemotaxis.<br>and ROS production.<br>• Evaluation of TNF-α production.	• Decreased chemotaxis and ROS production.<br>• Decreased TNF-α (mRNA and protein) levels.	↓	[156]
PMN from human healthy subjects and psoriasis patients.	PMNs were activated with LPS and treated with CBD (10 μM).	• Evaluation of NET production.<br>• Evaluation of MPO.	• Inhibition of NET production and cfDNA.<br>• Reduction of MPO levels.	↓	[139]

PMN from human healthy subjects.	PMNs were activated with fMLP and treated with CBD (from 0.1 μM to 1000 μM).	• Evaluation of chemotaxis.<br>• Evaluation of oxygen consumption.<br>• Evaluation of ROS production.	• Decreased chemotaxis.<br>• Decreased of oxygen consumption.<br>• Increased ROS production.	↓↑	[154]
8/9-weeks-old male nude rats (Hsd:RH-Foxn1rnu) (260–302 g).	Skin inflammation model was induced by irradiation with UVA/UVB, CBD (120 mg/kg) was administered topically together with UV radiation, at different times.	• Evaluation of ROS production.<br>• Evaluation of TNF-α production.<br>• Evaluation of NF-κB and CB1R expression.	• Reduction of ROS production.<br>• Reduction of TNF-α levels.<br>• Reduction of NF-κB and CB1R expression.	↓	[164]
Male adult Dunkin-Hartley guinea pigs (250-350 g).	Pulmonary inflammation was induced by aerosolised solution of LPS, CBD (10-50-100 mg/kg) was administered i.p. 1 hour before LPS treatment.	• Evaluation of PMNs infiltration in the airways.	• Decreased the PMNs infiltration in airways.	↓	[160]
PMNs from healthy subjects.	Resting and activated cells treated with CBD.	• Evaluation of COX 1/2 and PGE_2.	• Inhibition of expression of COX-1 and COX-2 mRNA in activated PMNs.	↓	[26]

IgE = immunoglobulin E; HSA = human serum albumin; CBD = cannabidiol; LPS = lipopolysaccharide;; MMP9 = metalloproteinase 9; MPO = myeloperoxidase; GFAP = glial fibrillary acidic protein; HBMEC = human brain microvascular endothelial cell; BBB blood brain barrier; LDH = lactate dehydrogenase; V-CAM1 = vascular cell adhesion protein 1; VEGF = vascular endothelial growth factor; ROS = reactive oxygen species; IL (interleukin)- 1Ra/8/16/32/1A/6/27/2/1β/4/17a/10/12p70; UDP = uridine diphosphate; ATP = adenosine triphosphate; IFN-γ/β = interferon-γ/β; TNF-α = tumor necrosis factor α; NO = nitric oxide; NOs = nitric oxide synthase; EAE = experimental autoimmune encephalomyelitis; CNS = central nervous system; CXCL1/9/10 = C-X-C motif chemokine ligand 1/9/10; ; I-TAC = interferon-inducible T cell alpha chemoattractant; M-CSF = macrophage colony stimulating factor 1; MCP-1 = monocyte chemoattractant protein-1; CCL2/5 = C-C motif chemokine ligand 2/5; NF-κB = nuclear factor kappa-light-chain-enhancer of activated B cells; G-CSF = granulocyte - colony stimulating factor; GM-CSF = granulocyte-macrophage colony-stimulating factor; MIF = macrophage migration inhibitory factor; TLR 1/3/4/9 = toll like receptor !/3/4/9; TGF- β1 = transforming growth factor β1; IP-10 = interferon gamma-induced protein 10; mTOR = mammalian target of rapamycin; COX 1/2 = cyclooxygenase 1/2; MCT = monocrotaline; MHCII = major histocompatibility complex class II; DSS = dextran sodium sulfate; cGAS-STING = cyclic GMP–AMP synthase-stimulator of interferon genes; NLRP3 = NLR family pyrin domain containing 3; HFC = high fat-cholesterol diet; STAT3 = signal transducer and activator of transcription 3; H_20_2 = hydrogen peroxide; TRPV2 = transient receptor potential cation channel subfamily v member 2; TREM2 = triggering receptor expressed on myeloid cells 2; GPR34 = G protein-coupled receptor 34; CR3 = complement receptor 3; PMNs = polymorphonuclear neutrophils; RANTES = regulated upon activation, normal T cell expressed and presumably secreted; CB1/2R = cannabinoid receptor 1/2; fMLP = N-formylmethionine-leucyl-phenylalanine; PMA = phorbol 12-myristate 13-acetate; NET = neutrophil extracellular traps; cfDNA = cell free DNA; I/R = ischemia/reperfusion; i.p. = intraperitoneally; N1/2 = neutrophils 1/2; UVA/UVB = ultra-violet light A and B; PGE2 = prostaglandin E2;
